# Magnetic Resonance Imaging Features of the Nigrostriatal System: Biomarkers of Parkinson’s Disease Stages?

**DOI:** 10.1371/journal.pone.0147947

**Published:** 2016-04-01

**Authors:** Lucie Hopes, Guillaume Grolez, Caroline Moreau, Renaud Lopes, Gilles Ryckewaert, Nicolas Carrière, Florent Auger, Charlotte Laloux, Maud Petrault, Jean-Christophe Devedjian, Regis Bordet, Luc Defebvre, Patrice Jissendi, Christine Delmaire, David Devos

**Affiliations:** 1 Department of Movement Disorders and Neurology, Lille University Hospital, Lille, France; 2 INSERM U1171, Faculty of Medicine, Lille University, Lille, France; 3 Department of Neuroradiology, Lille University Hospital, Lille, France; 4 Department of Small Animal Imaging Unit, Lille University, Lille, France; 5 Department of Medical Pharmacology, Lille University, Lille, France; University of Cincinnati, UNITED STATES

## Abstract

**Introduction:**

Magnetic resonance imaging (MRI) can be used to identify biomarkers in Parkinson’s disease (PD); R2* values reflect iron content related to high levels of oxidative stress, whereas volume and/or shape changes reflect neuronal death. We sought to assess iron overload in the nigrostriatal system and characterize its relationship with focal and overall atrophy of the striatum in the pivotal stages of PD.

**Methods:**

Twenty controls and 70 PD patients at different disease stages (untreated *de novo* patients, treated early-stage patients and advanced-stage patients with L-dopa-related motor complications) were included in the study. We determined the R2* values in the substantia nigra, putamen and caudate nucleus, together with striatal volume and shape analysis. We also measured R2* in an acute MPTP mouse model and in a longitudinal follow-up two years later in the early-stage PD patients.

**Results:**

The R2* values in the substantia nigra, putamen and caudate nucleus were significantly higher in *de novo* PD patients than in controls. Early-stage patients displayed significantly higher R2* values in the substantia nigra (with changes in striatal shape), relative to *de novo* patients. Measurements after a two-year follow-up in early-stage patients and characterization of the acute MPTP mouse model confirmed that R2* changed rapidly with disease progression. Advanced-stage patients displayed significant atrophy of striatum, relative to earlier disease stages.

**Conclusion:**

Each pivotal stage in PD appears to be characterized by putative nigrostriatal MRI biomarkers: iron overload at the *de novo* stage, striatal shape changes at early-stage disease and generalized striatal atrophy at advanced disease.

## Introduction

Parkinson’s disease (PD) encompasses various endophenotypes, ranging from slowly progressing, "pure" motor forms to rapidly progressing, extensive forms. There is a clear need for radiological biomarkers capable of predicting disease progression and thus assessing the efficacy of forthcoming disease-modifying treatments. PD is characterized by degeneration of the dopaminergic nigrostriatal pathway, beginning within the substantia nigra (SN). Dopaminergic neurons are exposed to high levels of reactive oxygen species (ROS) produced by the dopamine metabolism [1]. By catalysing the formation of ROS from the byproducts of oxygen consumption, high levels of labile iron can also affect neuronal functions by damaging cell components or oxidation-prone signalling molecules [1]. Although iron accumulation in the SN has been repeatedly demonstrated in pathology studies [2,3], the extent of iron accumulation in the putamen, pallidum and caudate nucleus (CN) is subject to debate in magnetic resonance imaging (MRI) studies [4,5]. MRI is indeed the easiest and most frequently radiological employed method for detecting iron content, since the metal induces magnetic field inhomogeneity, nuclear relaxation and spin dephasing via susceptibility effects. Most studies are based on a T2* sequence and report the R2* value (1/T2*) as a measure of total non-heme iron content. Another MRI sequence is employed to measure the reduced transverse relaxation rate (RR2), which is predominately affected by ferritin iron, separately from hemosiderin spin dephasing effect [6]. Many studies based on these two MRI methods have confirmed higher R2* and/or RR2 in the SN in PD patients than in controls [2–11]. However, the relationship of R2* with the pivotal clinical stages in PD must be specified. There is also a need to better understand the possible extension of iron overload to the basal ganglia and its interplay with atrophy (i.e. whether iron overload is the cause or simply a consequence of neurodegeneration), with control for the effect of age and cognitive disorders.

Volumetric studies of the putamen, pallidum and CN in PD have yielded contradictory results with various volume analysis methods. Some reported caudate and/or putamen atrophy using regions of interest (ROI) manual segmentation [12,13], sometimes correlated with cognitive decline with radial distance method [14]. Others, using voxel-based morphometry, did not detect any volume changes in these structures [5]. Moreover, overall volume analysis provides limited information on atrophy, which varies across the anatomic structure. Shape analysis can now detect more subtle focal changes in subcortical regions [15,16]. Shape analysis studies of early-stage PD have evidenced greater surface contraction (and thus atrophy) in the caudal putamen and the head and dorsal body of the CN [17,18]. However, shape changes at the pivotal stages of PD and the interplay with iron levels have not previously been investigated.

Hence, the objectives of the present study were to (i) measure R2* values in the SN, CN and putamen, and assess their relationship with overall and focal atrophy of the striatum in the pivotal stages of PD, (ii) establish whether these parameters could be biomarkers of disease progression.

## Methods

### Participants

Between October 2009 and May 2013, we enrolled healthy controls and PD patients (diagnosed according to Gibb's criteria and the Parkinson's Disease Society criteria [19]) at pivotal stages of PD: (i) *de novo* disease, defined as less than 1.5 years of disease progression since the initial sign and no antiparkinsonian treatments; (ii) early-stage disease, defined as less than 3 years of disease progression, with antiparkinsonian treatment but no L-dopa-related motor complications (LDRMCs); these early stage PD patients had a second MRI two years later for a longitudinal study (iii) advanced disease, defined as 10 to 15 years of disease progression with severe LDRMCs. In order to observe the effect of the disease process *per se*, we controlled for the effect of age and cognitive disorder (i.e. age matched groups and the lack of severe cognitive disorders). The main exclusion criteria for all participants were the presence of dementia (diagnosed according to the Movement Disorders Society criteria [20], psychiatric disorders as assessed in a semi-structured interview with a psychiatrist (in accordance with the Diagnostic and Statistical Manual of Mental Disorders [21]), disorders of iron metabolism, other metabolic disorders, radiological abnormalities (such as focal lesions or widespread ischemic changes in the basal ganglia) and other serious medical conditions. All clinical investigations were performed in accordance with the tenets of the Declaration of Helsinki. All participants provided written, informed consent to participation prior to inclusion. The protocol was approved by the independent ethics committee at Lille University Hospital (French national reference number: 2008−006842−25). As dementia was an exclusion criterion, all participants had the capacity to provide their consent.

Motor handicap was rated (according to the UPDRS motor score) under "on-drug" and "off-drug" conditions (i.e. at least 12 hours after the last dose of dopaminergic medication) in early and advanced-stage PD patients and necessarily only under "off-drug" condition for the *de novo* patients.

### Study flowchart

All participants underwent baseline MRI, in order to determine R2* values, volume and shape modifications. We predefined at the start of the study to enroll a greater number of early stage patients than in the three other groups for a second MRI acquisition two years later, with a focus on the evolution of R2* values.

**Fig 1:**
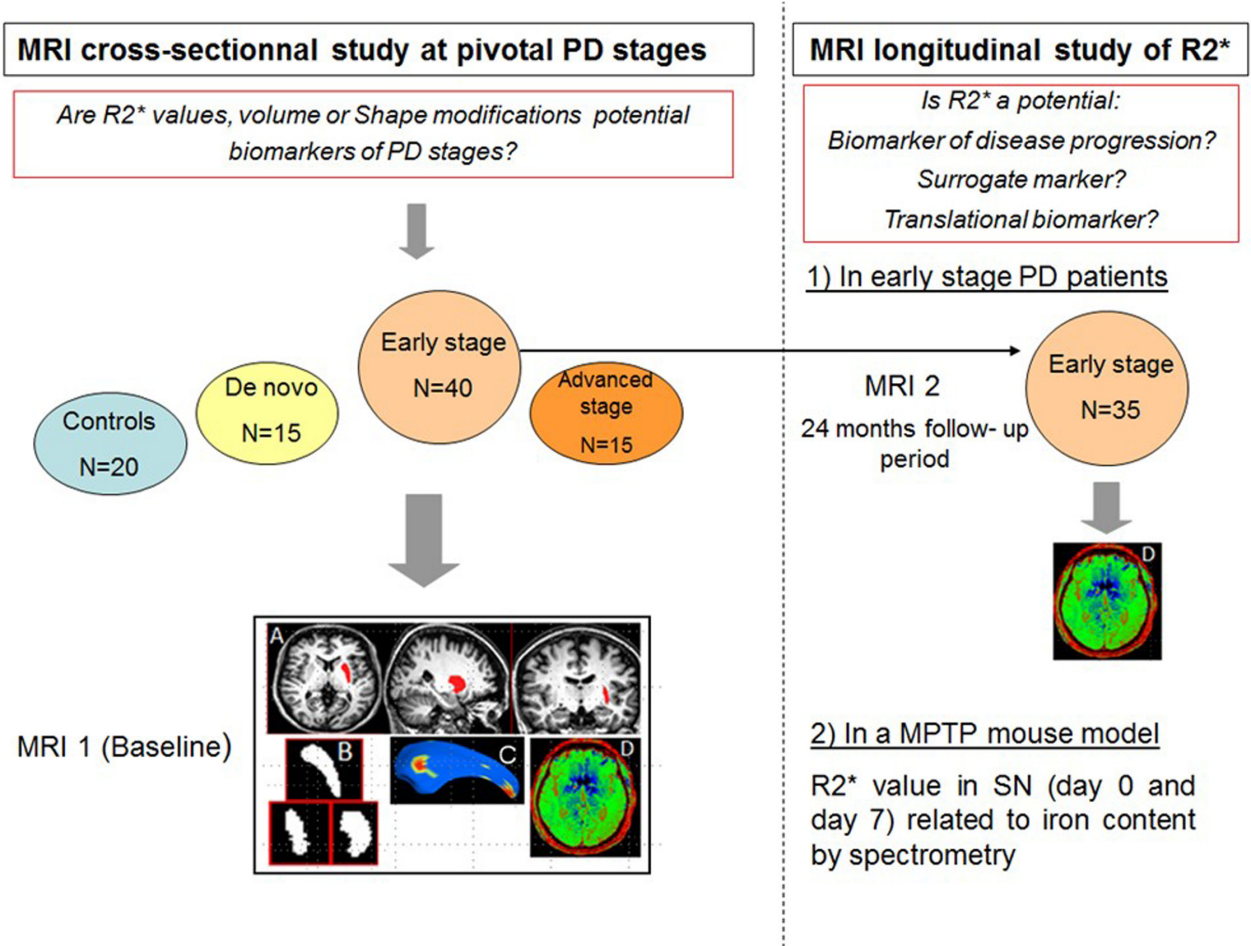
The study flowchart. MRI = Magnetic resonance imaging. PD = Parkinson’s disease. SN = Substantia nigra. MPTP = 1-methyl-4-phenyl-1,2,3,6-tetrahydropyridine. Delimitation of Region of interest (ROI) by a semi-automatic segmentation (A), extraction of ROI 3D mask to calculate the volume, achieve shape analysis (C), and registration on the T2* map sequence (D) and obtain mean R2* value of the ROI.

Lastly, we used the 1-methyl-4-phenyl-1,2,3,6-tetrahydropyridine) (MPTP) mouse model to determine whether the higher *in vivo* R2* value in the SN is related to acute iron overload and thus may have value as a translational biomarker (Fig 1).

### Magnetic resonance imaging

All MRI datasets were acquired with a 3 Tesla (T) machine (Achieva, Philips Medical Imaging. Best, The Netherlands) equipped with an eight-channel head coil. A 3D, T1-weighted, fast-field echo sequence (TR (Repetition Time): 9.8 ms, TE (Echo Time): 4.6 ms, flip angle: 8°; matrix size: 256 × 256 x 160; field of view: 256 × 256 x 160; voxel size: 1 × 1 × 1 mm^3^) and a and a T2*-weighted FFE sequence with 15 different echo times (TR: 1819 ms; TE: from 3.7 ms to 50.29 ms; flip angle: 80°; matrix size (2stacks): 115 x 95 x 17; field of view: 230 x190 x 34 mm3; voxel size: 2 x 2 x 2 mm^3^)) were performed for each study participant.

#### Semi-automatic segmentation of subcortical structures and T2* sequence processing

ROIs (left and right putamen and caudate nucleus) were anatomically delineated using a semi-automatic approach on the3D T1-weighted images. The initial step was an automatic brain segmentation with Freesurfer software (http://surfer.nmr.mgh.harvard.edu/). In order to rule out segmentation errors, ROIs were visually inspected and (if required) manually corrected by an investigator blinded to the participant's condition. T2* maps were generated with the relaxometry routine in the Philips Research Imaging Development Environment (PRIDE^®^, Philips Medical Imaging). A mono-exponential trend-line was fitted with this equation: y = Kexp(–TE/T2*), where y is the MR signal intensity, K is a constant and TE is the echo time. Head motion correction was performed by rigid registration (3 translations and 3 rotations) of echoes. The first echo was used as reference.

The T1 sequence was then registered to the T2* space and the T2map sequence using the rigid body registration provided by the Advanced Normalization Tools package (http://picsl.upenn.edu/software/ants/). The transformation matrix was then applied to subcortical ROIs. The output of this registration step was checked by an expert radiologist and (if required) manually corrected. Lastly, the volume (in mm^3^) normalized to individual intracranial volume and the mean R2* (1/T2*) time (in ms) were calculated for each ROI.

As we could not obtain a reliable 3D mask for the SN on the T1 image, a planar ROI was drawn on the right and left SN (comprising both pars compacta and reticulata) on the sixth echo time of the T2* sequence because it had the clearest SN signal and thus the most reliable borders. The SN was defined on an axial section as a hypo intensity band between the red nucleus and cerebral peduncle. To avoid to include the subthalamic nucleus, the delimitation was started at the level where the red nucleus had the largest radius as described by Du et al [7] The investigators blinded to the participants condition performed two delimitations for each body side for intra-rater reliability, and the mean R2* value inside this area was calculated.

#### Shape analysis

Shape analysis was performed using the Spherical Harmonics Point Distribution Models (SPHARM-PDM) toolbox [15]. Each ROI is modelled by a 3D spherical harmonic function. The surface meshes and corresponding spherical parameterization are then computed. The first-order spherical harmonic is used to align the spherical parameterization with the structure's main axes. Each ROI is then closed (with a closing factor of 3 mm or less), smoothed, binarized and converted into surface meshes. The latter are mapped onto a sphere and spherical harmonic coefficients are computed. The spatial locations of the vertex in the various groups were then compared to determine if there were shape modifications such as surface contraction (atrophy) or expansion (hypertrophy).

The magnitude of the projection of these different vectors is then shown on a colour map. The color coded by red are negative distance (shrinking below the mean surface of normal controls)

### The 1-methyl-4-phenyl-1,2,3,6-tetrahydropyridine (MPTP) mouse model

We analyzed the relationship between the MRI R2* value and iron overload in the SN in 10 saline-treated mice and 10 MPTP-treated mice (5-month-old male C57Bl/6J mice, weighting 28–30 g; Janvier Le Genest St Isle, France) (MPTP 4 x 20 mg/kg i.p. over 24h).

All experimental procedures were carried out in strict accordance with the recommendations in the Guide for the Care and Use of Laboratory Animals of the Ministry of Health with current French and international guidelines (French Governmental Decree no. 87–848 issued on October 19th, 1987 by the French Ministry of Agriculture and Forestry’s Veterinary Service for Animal Health and Protection). The experimental protocol was reviewed and approved by the Bioethic Committee of North of France CEEA75 (Permit Number: CEEA75- 102012). Mice were quarantined for ≥7 days before study initiation and daily checked for healthy status and cares. If suffering limit point was detected, mouse was sacrificed by letal dose injection of pentobarbital. Ten mice in each group were utilized to minimize the number of experimental animals needed while ensuring the validity of statistical power.

#### Mice behaviour test

The animals' spontaneous motor activity was recorded seven days after MPTP intoxication over a 10-minute period in an actimeter equipped with Actitrack analytical software (Panlab, Barcelona, Spain). The transparent Plexiglas open field was equipped with two frames of infrared beams for measuring horizontal motor activity (distance travelled, speed and type of movement) and vertical motor activity (rearing).

#### *In vivo* mice MRI

During acquisition, animals were anesthetized with a mixture of air and isoflurane concentrate between 1–2% depending of the breathing. Mouse head was fixed with ear plug and tooth bar to prevent head movement during acquisition. The mouse body temperature was maintained at 37°c using warm water circulating inside the bed. Animal breathing was monitored with a sensor pillow placed on the abdomen.

The MRI experiments were performed just after behaviour test at 7 Teslas in a horizontal bore magnet (Bruker, Biospec, Ettlingen, Germany). For the cerebral imaging, a volume coil (internal diameter 72mm) was used for radio frequency emission, and a surface coil is placed on the animal skull for the signal reception.

A sagittal T2 TurboRARE (Rapid Acquisition with Relaxation Enhancement) spin echo sequence was only performed to get anatomical information in order to position the slices for the imaging of subtantia nigra. Sequences were the following: TR/TE = 5000 / 33ms, matrix size 256 x 256 pixels, field of view 20 x 20mm, 10 contiguous slices of 0.5mm thickness were acquired.

The assessment of T2* decay was performed on a unique axial slice positioned on subsantia nigra region. T2* Multi Gradient Echo sequence parameters were the following, TR = 1500ms, the echo times values were incremented of 5ms and comprised between 4ms and 60ms. The geometric parameters were: a square FOV of 20mm encoding by a 256 square matrix, a slice thickness of 0.4mm.

ROIs were first manually drawn on axial T2* images to delineate SN. Then SN T2* value was assessed in using an mono-exponential curve described by 12 echoes. Post-treatment images of T2* decay were performed on Paravision 5.0 (Bruker, Germany).

#### Histological brain analysis

After MRI experiment mice were deeply anaesthetized with sodium pentobarbital and transcardially perfused with 4% paraformaldehyde in 0.1 M phosphate buffer for tissue fixation (pH 7.4). Brain section analyses were performed on 6-micrometre-thick coronal sections prepared from the SN/ventral tegmental area using a microtome (Leica, Nussloch, Germany). Three sections were taken (-2.92 mm, -3.16 mm and -3.4 mm, relative to the bregma), as previously described [22], and the number of TH-positive neurons per sample was counted under a light microscope (400x magnification) by a highly trained scientist (CL) who was blinded to the animals' treatment status.

#### Iron brain quantification

SNc samples were heated in 10% nitric acid buffer for mineralization and iron assay was performed by furnace atomic absorption spectrophotometry (Perkin-Elmer AA800).

### Statistical analysis

#### R2* value, volume and MPTP mouse model data

In view of the sample size and the skewed data distributions for most of the criteria, all quantitative variables are expressed as the median [interquartile range]. We used an analysis of variance to estimate intergroup differences. For non-normally distributed data, the robustness of the results was checked after log transformation. A Bonferroni *post hoc* analysis was performed. Spearman correlation was applied between clinical and radiological parameters.

#### Shape analysis

The SPHARMPDM toolkit's routine for multivariate analysis of covariance [15] was used in the shape analysis. The 3D spatial coordinates were treated as multivariate response variables at each surface point and then adjusted for age and gender (by general linear modelling and permutation-based testing of the local Hotelling trace). The distance and direction between group mean surfaces at each pair of corresponding points were computed in order to generate a vector field map that representing the displacement between PD and controls. P-values were adjusted with the Benjamini-Hochberg false discovery rate procedure. The Wilcoxon signed-rank test was used to compare two related samples (i.e. repeated measurements). All statistical tests were two-tailed. The threshold for statistical significance was set to p<0.05. All statistical analyses were performed with SPSS software (version 22).

## Results

### Cross-sectional study of pivotal PD stages and controls

A total of 90 subjects were recruited into the study, 15 de novo, 40 early stage 15 advanced stage PD patients and 20 controls. Demographic and clinical features of the subjects are described in Table 1.

**Table 1:** Demographic and clinical characteristics of PD patients and controls.

	Controls	PD patients			
		*de novo*	Early stage	Advanced stage	p value
Number of patients	20	15	40	15	
Age at study entry (years)	59 [57–65]	64 [59–67]	60 [52–64]	59 [52–65]	F_(3, 86)_ = 1; p<0.2
Gender (females/males)	9/11	7/8	15/20	7/8	ns
Time since diagnosis (years)	-	1 [0.5–1.5]	2 [2–3]	12 [10–14]	F_(2, 67)_ = 183; p<0.0001
Hoehn and Yahr stage)	-	1.5 [1–1.8]	2 [1.6–2]	2.5 [2–2.8]	F_(2, 67)_ = 28; p<0.0001
Most severe side (right/left)	-	8/7 (6 uni)	16/19	8/7	ns
UPDRS motor score off dopa	-	16 [14–18]	26 [24–35]	42 [38–49]	F_(2, 67)_ = 25; p<0.0001
UPDRS motor score on dopa	-	-	16 [14–20]	24 [17–28]	F_(1, 53)_ = 4.6; p = 0.036
Daily L-dopa dose equivalent (mg)	-	0	300 [150–532]	1065 [796–1291]	F_(1, 53)_ = 57; p<0.0001
Patients on L-dopa (n)	-	0	17	14	ns
Patients on dopaminergic agonists	-	0	30	12	ns
Patients on L-dopa and agonists (n)	-	0	13	10	ns
Mattis Dementia Rating Scale score	142 [139–144]	141 [139–143]	139 [138–140]	139 [133–143]	F_(3, 86)_ = 0.5; p<0.7

Quantitative variables are quoted as the median [interquartile range]. “uni” means unilateral PD = Parkinson’s disease.

#### Iron overload in the nigrostriatal system (Table 2, Fig 2A)

There were significant intergroup differences in the R2* value for the SN, the CN and the putamen on both sides of the body. A Bonferroni *post hoc* analysis revealed a significant higher R2* value on both sides (i) in the SN, the putamen and the CN in *de novo* PD patients, relative to controls; (ii) in the SN in early-stage patients, relative to *de novo* patients; and (iii) in the SN in advanced-stage patients, relative to *de novo* patients. There were no significant differences between early-stage and advanced-stage patients for the SN, the putamen and the CN. Six *de novo* patients had unilateral Parkinson syndrome and all displayed abnormal R2* values on both sides of the body. Forty-eight of the 70 PD patients had the highest R2* value in the SN that was contralateral to the most clinically affected body side. There was no correlation between the ROIs R2* values and UPDRS III score or L-Dopa equivalence dose.

**Fig 2:**
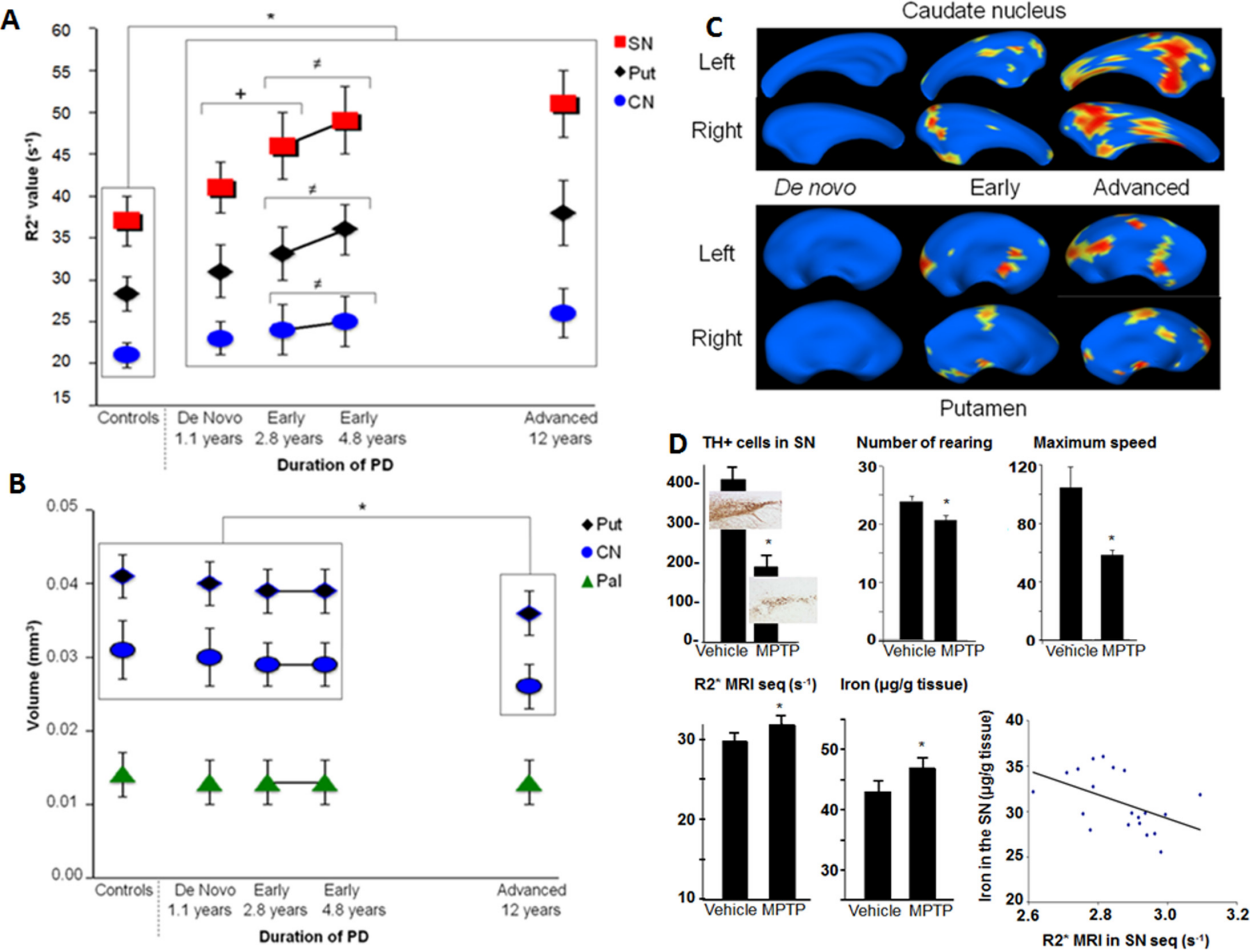
(A): R2* values in the nigrostriatal system at different stages of PD; Fig (B): Striatal volumes at different stages of PD; (C): Shape analysis of caudate nucleus and putamen at different stages of PD relative to controls. In blue no statistical difference p>0.05, from yellow to read decrease of p value from p<0.05 (p value of read surface< pvalue of yellow surface<0.05). Yellow and read surface correspond to surface contraction and thus atrophy; **(D): Iron overload in the MPTP mouse model of PD.** Mice were treated with MPTP (or not; n = 10 per group). Data are presented as the mean and SEM. Immunohistochemistry of tyrosine hydroxylase (TH) stained sections of the right substantia nigra (SN), illustrating the level of degeneration after MPTP intoxication. TH+ cell counts of both sides of the SN. *: Wilcoxon test: p<0.0001. Total iron levels in the SN, as measured by atomic absorption spectrometry. *: p = 0.035. R2* multiple-echo spin echo value (7 T MRI) in the SN (MRI performed *in vivo* prior to sacrifice and spectrometry measurements) (R2* = 1/T2*(ms-1) x 103) *: p = 0.001. Motor handicap scores (measured in a 10-minute actimetry test). Number of rearings *: p = 0.004. Maximum speed: *: p = 0.03.

**Table 2:** Radiological characteristics of controls and PD patients (volume, and R2* value).

	Controls	*De novo*	Early-stage	Advanced-stage	p value
Iron overload: R2* value from MRI (s^**-1**^**)**					
Left substantia nigra	37.7 [36–39]	42.5 [39–43]*	46.7 [43–52]**	50.2 [45–52]***	F_(3,86)_ = 26; p<0.0001
Right substantia nigra	36.8 [35–39]	41.6 [37–46]*	475 [43–51]**	47.8 [45–52]***	F_(3,86)_ = 14; p<0.0001
Left caudate nucleus	21.9 [21–22]	23.5 [22–24]*	**24.9 [22–25]**	**24 [22–26]**	F_(3,86)_ = 6; p = 0.001
Right caudate nucleus	21.4 [20–22]	23.3 [22–25]*	**23.1 [22–25]**	**24.7 [22–27]**	F_(3,86)_ = 7; p = 0.0001
Left putamen	28 [27–28]	32.2 [31–35]*	**32.5 [31–35]**	**33 [30–38]**	F_(3,86)_ = 17; p<0.0001
Right putamen	28.6 [28–30]	30.9 [31–35]*	**31.4 [32–35]**	**36.1 [32–38]**	F_(3,86)_ = 13; p<0.0001
Left pallidum	38.6 [37–43]	41.7 [37–45]	39 [37–41]	40.8 [39–44]	F_(3,86)_ = 0.5; p = 0.7
Right pallidum	38 [36–40]	39.9 [36–45]	40 [37–44]	40.1 [37–44]	F_(3,86)_ = 0.8; p = 0.5
**Atrophy: 3D volume on MRI (mm**^**3**^**.10**^**3**^**)**					
Left caudate nucleus	30.6 [27–33]	3.14 [2.8–3.3]	2.9 [2.6–3.9]	**2.54 [2.2–2.9]**	F_(3,86)_ = 8; p = 0.0001
Right caudate nucleus	30.5 [28–33]	3.00 [2.6–3.3]	2.8 [2.6–2.9]	**2.45 [2.3–2.6]**	F_(3,86)_ = 13; p<0.0001
Left putamen	40.1 [38–43]	3.98 [3.9–4.3]	3.96 [3.8–4.1]	**3.63 [3.4–3.8]**	F_(3,86)_ = 11.6; p<0.0001
Right putamen	40.1 [38–43]	4.09 [3.9–4.3]	3.99 [3.8–4.3]	**3.64 [3.2–3.8]**	F_(3,86)_ = 6; p<0.001
Left pallidum	14 [13–15]	1.38 [1.2–1.4]	1.26 [1.1–1.5]	1.30 [1.2–1.4]	F_(3,86)_ = 0.5; p = 0.6
Right pallidum	13.1 [13–14]	1.37 [1.1–1.4]	1.44 [1.1–1.6]	1.30 [1.2–1.4]	F_(3,86)_ = 0.4; p = 0.7

Quantitative variables are quoted as the median [interquartile range]. PD = Parkinson’s disease. **p-value in the right column is the significant intergroup difference.** *Post hoc* analysis of Bonferroni

* means significant (p<0.05) higher R2* value in the SN, the putamen and the CN in *de novo* PD patients, relative to controls

** means significant (p<0.05) higher R2* value in the SN in early-stage patients, relative to *de novo* patients

*** means significant (p<0.05) higher R2* value in the SN in advanced-stage patients, relative to *de novo* patients.

#### Striatal volume (Table 2, Fig 2B)

Significant intergroup differences in volume were observed for the CN and the putamen on both sides of the body. *Post hoc* analyses showed that the volumes of the CN and the putamen were significantly lower in advanced-stage patients than in the three other groups. There were no significant differences between controls, *de novo* patients and early-stage patients.

No significant differences in R2* values or volume were observed outside the nigrostriatal system, including the pallidum (Table 2) and the motor cortex (data not shown).

#### Striatal shape analysis (Fig 2C)

We observed significant intergroup differences in shape, as evidenced by surface contraction of the CN and the putamen in early-stage patients and advanced-stage patients relative to controls and *de novo* patients, and a significance difference between early-stage and advanced-stage patients. There was no significant difference between controls and *de novo* patients.

### Longitudinal study of early-stage PD patients (Fig 2A)

We performed a longitudinal analysis in 35 of the early-stage PD patients focusing on R2* as it seems to be a potential early biomarker. All these patients displayed a worsening of the clinical handicap (mean ± SD UPDRS motor score: 16±9 at baseline and 20.7±15 at two years; mean change: 4.7; p = 0.001) and a concomitant increase in the R2* on both sides of the body with a progression of 6% in the SN (mean change: +0.3; range: +0.01 to +0.7; z = -5.5; p<0.001), 8% in the putamen (mean change: +0.29; z = -6.2; p<0.001) and 4% in the CN (mean change: +0.1; z = -4.5; p<0.001) after 2 years. There was a moderate but statistically significant correlation between the change in R2* in the SN and the change in the UPDRS motor score (Spearman correlation: r^2^ = -0.5; p = 0.03).

### Relationship between the R2* values and iron overload in the acute MPTP mouse model (Fig 2D)

Seven days after the acute intoxication with MPTP, the mice displayed motor handicap (i.e. fewer rearings and a lower mean speed) and a 50% reduction in the number of tyrosine-hydroxylase-positive neurons. A significantly higher R2* value in the SN (measured *in vivo*) was associated with a significant higher level of iron (as measured by spectrometry) in the MPTP mice (relative to control animals). The R2* values and the iron level were significantly correlated (Spearman correlation coefficient: r^2^ = -0.52; p = 0.019). A non-significant trend towards higher R2* values was noted for the putamen and CN.

## Discussion

Since PD has a slow clinical progression, we combined a cross-sectional analysis to compare R2* values, shape and volume between different PD stages with a longitudinal study to follow the R2*value modification with disease progression. Although a long-term longitudinal analysis of all MRI features would doubtless have provided interesting information, it would have required at least 10 to 15 years of follow-up. Moreover, one of our study's strengths relates to the fact that we controlled for the effect of age, since physiological levels of iron increase with age in the putamen, the CN and the cortex according to pathology studies [23] and MRI measurements (24).

Concerning R2* values, volume and shape analysis, our results showed that iron overload affects the whole nigrostriatal system (and predominantly the SN) from the *de novo* stage of PD onwards. Higher iron content in the SN even preceded the onset of motor symptoms in the 6 *de novo* patients with unilateral Parkinson syndrome with high R2* values on both body sides. The early high R2* values in the SN in our study is in accordance with all studies to date [4,7,8–11]. However, our findings for the striatum reveal that iron overload appears to be more limited in this structure. Although most studies reported a mild striatal iron overload (4, 7, 9, 10), others did not find any differences [5]. Moreover, focal atrophy of the striatum measured in our study by shape analysis, occurred in early-stage disease and preceeded mild but more diffuse atrophy of the CN and putamen in advanced PD. This finding confirmed recent shape analyses in early-stage PD, which showed that surface contraction was greater for the caudal putame [17,18] and the head and dorsal body of the CN [17]. Concerning global volumetric data, our results are similar to studies who reported atrophy of the putamen [12], of the caudate [13] or of the caudate only in late-stage, demented PD patients [14], However others did not find any significant volume differences for the putamen, pallidum and CN in PD [5]. Even if it is difficult to establish a direct causality, these data seem to show that iron overload appears at earlier PD stages than focal or generalized atrophy, and thus could be involved in disease progression and is not a simple consequence of degeneration.

Our results of the longitudinal study for 35 patients confirmed R2* rate of progression for the nigrostriatal system observed in a longitudinal study of 14 early-stage PD patients of much the same age and rate of disease progression [9]. Ulla *et al*. observed a mean increase of 9% in the SN and 11% in the caudal putamen, after three years of disease progression [9], this compares with our values of 6% in the SN and 8% in the putamen, after two years of disease progression. Our results also highlighted the small but significant progression of 4% in the CN. The most recent longitudinal study also showed higher R2* values in the SN, the CN and the anterior part of the pallidum after two years [24]. Interestingly, the involvement of the pallidum and the CN might be related to the greater age of the study population (median: 73 years) and the presence of cognitive disorders [25]. However, the lack of a control group in our longitudinal R2* data does not rule out the possibility that higher R2* values are age-related and not only due to PD progression.

Lastly, disease progression and iron overload were not linearly correlated. Iron overload worsened rapidly over the first 3–5 years and then more slowly in advanced disease. Overall atrophy of the striatum might also interfere with the measurement of iron content. The non-linear progression of iron overload might explain the lack of correlation between motor handicap and iron content in the SN in cross-sectional studies [8,9]. As expected, our longitudinal study of the first years of disease evidenced a higher degree of correlation, which might be of interest for clinical trials. However, one must bear in mind that only free, labile, ferrous iron (Fe^2+^) and not bound ferric iron, Fe^3+^ has a harmful effect on the brain. Although the R2* MRI sequence only measures Fe^3+^, the latter is in equilibrium with Fe^2+^and R2* is an indirect or delayed measurement of the harmful effect of free labile iron overload.

However, our useful experiments in the MPTP mouse showed a correlation between the R2* MRI sequence and total iron, and thus confirmed previous *ex vivo* data [26] and the reliability of MRI results. Moreover, *in vivo* iron overload in the SN was observed by MRI in MPTP mice seven days after intoxication by MPTP, comforting our MRI results in PD patients with early increase R2* values. You et al. (2015) [27] also revealed that increased nigral iron content exacerbates oxidative stress and dopaminergic neuronal death in MPTP induced PD, demonstrating that R2* is an early, sensitive biomarker with translational potential.

The main limits of our study concerns the various image processing methods employed for ROI delimitation (VBM, radial distance, and manual or semi-automatic segmentation), which also in part explains the controversial results of volumetric studies. Manual segmentation is subject to inter- and intra-ratter variability, while automated segmentation is not yet fully reliable [28]. We applied a semi-automatic approach and our present results agree with a previous study that used the same image processing technique showing atrophy of the putamen in 16 patients with early PD (12.5%) and eight patients with advanced PD (26.5%) [12]. We were also not able to segment the SN on a conventional 3T T1 dataset. It has been possible to clearly delineate the SN on a 3D T2* dataset with a 7 T MRI; showing controversial results with either a greater [29] or smaller (29) SN volume in PD patients and irregular borders in the lateral SN [30]. Easy, efficient volume processing is therefore a prerequisite for assessing the potential influence of volume on iron measurements within the SN.

## Conclusion

We used three different MRI methods (R2*, shape analysis and volumetry) to characterize pivotal stages of PD. When the first signs of PD appear, the rate of disease progression and the impact of disease-modifying strategies could be assessed by monitoring the progression of iron overload in the SN (via R2*) and the appearance of focal atrophy of the CN and putamen (via shape analysis). Later PD stages could be characterized by determining focal and overall atrophy of the striatum in shape and global volume analyses. The long-term clinical value of these putative surrogate biomarkers must now be established longitudinally in an independent population as a function of disease stage.
